# Application of Network Analysis to Flow Systems with Alternating Wave Channels: Part A (Pressure Flows)

**DOI:** 10.3390/polym11091488

**Published:** 2019-09-12

**Authors:** Christian Marschik, Marius Dörner, Wolfgang Roland, Jürgen Miethlinger, Volker Schöppner, Georg Steinbichler

**Affiliations:** 1 Institute of Polymer Extrusion and Compounding, Johannes Kepler University Linz, 4040 Linz, Austria; wolfgang.roland@jku.at (W.R.); juergen.miethlinger@gmail.com (J.M.); georg.steinbichler@jku.at (G.S.); 2 Kunststofftechnik Paderborn, Universität Paderborn, 33098 Paderborn, Germany; marius.doerner@ktp.uni-paderborn.de (M.D.); volker.schoeppner@ktp.uni-paderborn.de (V.S.)

**Keywords:** wave-dispersion screw, modeling and simulation, polymer processing, extrusion, network theory

## Abstract

Wave-dispersion screws have been used industrially in many types of extrusion processes, injection molding, and blow molding. These high-performance screws are constructed by replacing the metering section of a conventional screw with a melt-conveying zone consisting of two or more parallel flow channels that oscillate periodically in-depth over multiple cycles. With the barrier flight between the screw channels being selectively undercut, the molten resin is strategically forced to flow across the secondary flight, assuring repeated cross-channel mixing of the polymer melt. Despite the industrial relevance, very few scientific studies have investigated the flow in wave-dispersion sections in detail. As a result, current screw designs are often based on traditional trial-and-error procedures rather than on the principles of extrusion theory. This study, which was split into two parts, was carried out to systematically address this issue. The research reported here (Part A) was designed to reduce the complexity of the problem, exclusively analyzing the pressure-induced flows of polymer melts in wave sections. Ignoring the influence of the screw rotation on the conveying characteristics of the wave section, the results could be clearly assigned to the governing type of flow mechanism, thereby providing a better understanding of the underlying physics. Experimental studies were performed on a novel extrusion die equipped with a dual wave-channel system with alternating channel depth profiles. A seminumerical modeling approach based on network theory is proposed that locally describes the downchannel and cross-channel flows along the wave channels and accurately predicts the pressure distributions in the flow domain. The solutions of our seminumerical approach were, moreover, compared to the results of three-dimensional non-Newtonian CFD simulations. The results of this study will be extended to real screw designs in Part B, which will include the influence of the screw rotation in the flow analysis.

## 1. Introduction

Single-screw extruders are the processing machines of choice for shaping polymers. They are used in various continuous manufacturing processes to produce finished or semifinished products such as films, pipes, profiles, and sheets. The most important machine components of single-screw extruders are a feeding system, a drive unit, a barrel, a temperature control system, and a screw. The latter is the most important element of the processing machine. Many single-screw extruders operate significantly below the maximum possible performance because of improper screw design. Over the past several decades, various high-performance screws have been developed to optimize the extrusion process for output and melt quality. This technical progress has gone hand-in-hand with extensive theoretical and experimental research [1,2,3,4]. Due to their complex geometries, however, some of these high-performance screws are still not properly understood, and their current designs offer potential for optimization.

**Figure 1:**
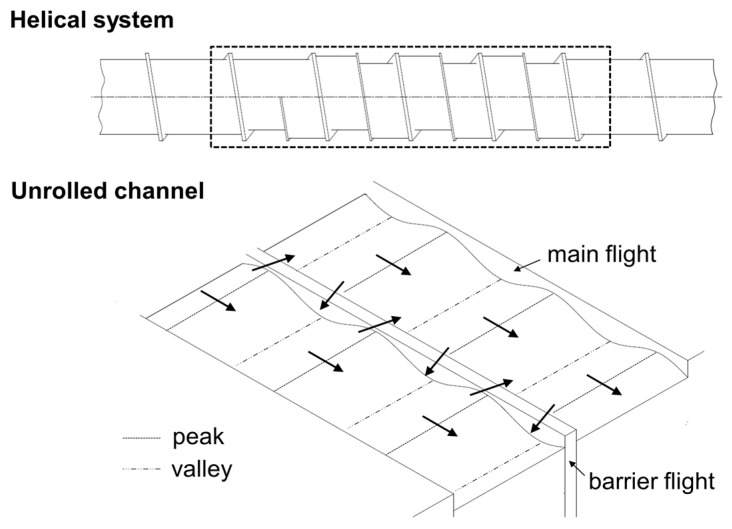
Schematic of a wave-dispersion zone with two flow channels.

This research investigated the operation of so-called wave-dispersion zones that were implemented to allow the extruder to operate at higher output rates without causing excessive temperatures and irregularities in the discharge. The term wave-dispersion zone refers to melt-conveying zones consisting of two or more parallel flow channels that oscillate cyclically in depth over a plurality of cycles, with alternating wave peaks and valleys, as illustrated in Figure 1. The wave cycles in adjacent channels are out of phase, i.e., the channel depth of one channel decreases while the other increases. The helical displacement between the channels is typically arranged such that a wave peak in one subchannel coincides with a valley in the other. The adjacent channels are separated by a barrier flight that is undercut relative to the main flight. In this manner, cross-channel mixing can be effectively facilitated, as material flowing down a channel toward a peak is forced to split its flow due to the decreasing cross-sectional area, with material portions remaining in the original channel and portions traveling across the barrier flight.

Several commercially available extruder screws have been designed according to wave technology [3]. The first wave-dispersion screws with multiple flow channels were developed by Kruder [5,6]. An enhanced design trademarked as an energy transfer (ET) screw was patented by Chung and Barr [7]. In this modified concept, the clearances of the screw flights were selectively interrupted to further promote cross-channel mixing between the channels. Other optimizations of the original design have been presented by Kruder [8], Medici et al. [9], Barr [10], and Womer et al. [11].

Despite their recognized performance, very few scientific analyses have examined the flow in wave-dispersion zones. Kruder and others [12,13] have carried out experimental studies to investigate the pumping capability of wave-dispersion screws. For these trials, the dual-channel screw design was largely superior to single-channel geometry in terms of output rates and melt temperatures. Similar extruder tests using an energy transfer screw were performed by Chung and Barr [14]. Fan et al. [15] and Perdiakoulias et al. [16] presented three-dimensional flow simulations of unrolled wave-dispersion sections. These numerical analyses visualized the flow pattern of the polymer melt, providing insights into the complicated nature of the flows. The results indicated an improved mixing performance of wave-channel systems. Surprisingly, this outcome was caused by the oscillating down channel velocity of the polymer melt rather than a repeated material transfer across the barrier flight. Somers et al. [17] expanded on the numerical analysis to examine the effect of thermal homogenization in helical energy transfer screw sections. In this study, cross-channel mixing was clearly detected as a result of the strategically positioned flight undercuts. The improved mixing capability of energy transfer sections was experimentally confirmed in Reference [18].

Physically, the flow of polymer melts in wave-dispersion zones can be divided into a down- and cross-channel component, both of which are governed by the rotation of the screw and the pressure distribution in the screw channel. The former causes a drag flow, and the latter gives rise to a pressure flow. Due to the shear-thinning behavior of polymer melts, all of these components are coupled via the dependency of the viscosity on shear rate. Since the channel depth is additionally a function of the downchannel coordinate, the shear rate changes at each position of the flow channel, and hence accurate flow analysis inevitably requires a local description of the flow mechanisms.

**Figure 2:**
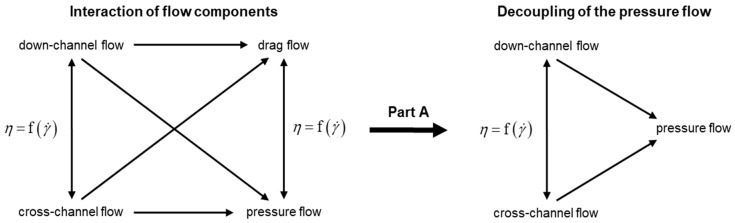
Network of flow components. To systematically simplify the physical process in single-screw extruders, the pressure flow was decoupled from the drag flow in this analysis (Part A).

This research was carried out in an effort to systematically increase the understanding of the flow in wave sections. Due to the complexity of the problem, the work was split into two parts. The research presented here (Part A) decoupled the network of flow components in a first step and focused exclusively on the analysis of pressure-induced flows, as indicated in Figure 2. By ignoring the drag flow, the complexity of the flow could be considerably reduced, and the results could be clearly assigned to the governing type of flow mechanism, providing a better understanding of the underlying physics. To consider the influence of screw rotation in the performance analysis, the study will be extended to superimposed drag and pressure flows in a following article (Part B).

In this analysis, experimental studies were performed on a novel extrusion die equipped with a dual wave-channel system (see chapter 2.2). The aim was to investigate the interaction between the downchannel and cross-channel flows and its effects on the conveying behavior of the wave section using various channel geometries, materials, and processing conditions. A seminumerical procedure based on network theory is presented that describes the governing flow mechanisms and accurately predicts the pressure distribution in the wave-dispersion zone. Avoiding time-consuming and traditional trial-and-error design procedures, this method can be used to systematically optimize the geometry of wave systems. Three-dimensional flow simulations were carried out to compare the results of our seminumerical approach to the solutions of a widely known numerical procedure.

## 2. Experimental Procedure

### 2.1. Materials

Two materials with different rheological behaviors were investigated: (i) a high-density polyethylene (HDPE) and (ii) a polypropylene random copolymer (PP-R). Table 1 provides an overview of the melt flow rates (MFR) of the materials.

**Figure 3:**
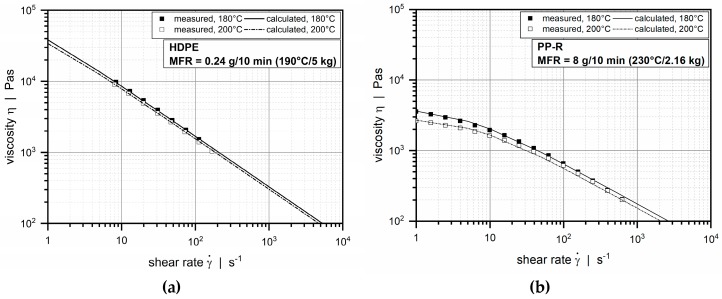
Comparison between experimental and calculated viscosity data at temperatures of 180 and 200 °C: (**a**) high-density polyethylene (HDPE) and (**b**) polypropylene random copolymer.

The viscosity of the higher viscous material was measured by means of a Göttfert Rheograph 25 high-pressure capillary rheometer (GÖTTFERT, Buchen, Germany), whereas the viscosity of the lower viscous material was determined using an Anton Paar MCR 302 plate–plate rheometer (Anton Paar, Graz, Austria). To describe the rheological flow behavior of the polymer melts mathematically, the experimental viscosity data were approximated by a temperature-dependent Carreau–Yasuda model [19,20]:

(1) $$\eta_{c}=a_{t}\eta_{\infty }+a_{t}\left(\eta_{0}-\eta_{\infty }\right){\left(1+{\left(a_{t}\lambda \dot{\gamma }\right)}^{a}\right)}^{\frac{n_{c}-1}{a}}$$

where η_0_ is the zero-shear viscosity, η*_∞_* is the infinite-shear viscosity, λ is the characteristic relaxation time, and *n*_c_ is the Carreau–Yasuda power law index. The parameter *a* describes the width of the transition between the Newtonian plateau and the shear-thinning region. The temperature-shift factor was calculated by

(2) $$a_{t}=\exp\left(-\alpha \left(T-T_{0}\right)\right)$$

where *α* is the temperature coefficient, and *T*_0_ is the reference temperature. Table 2 summarizes the Carreau–Yasuda parameter values for the HDPE and the PP-R. A comparison between calculated and experimental viscosity curves at temperatures of 180 and 200 °C is shown in Figure 3. The densities of the materials were measured as a function of pressure and temperature by means of a Göttfert Rheograph 25 high-pressure capillary rheometer (GÖTTFERT, Buchen, Germany).

### 2.2. Equipment

**Figure 4:**
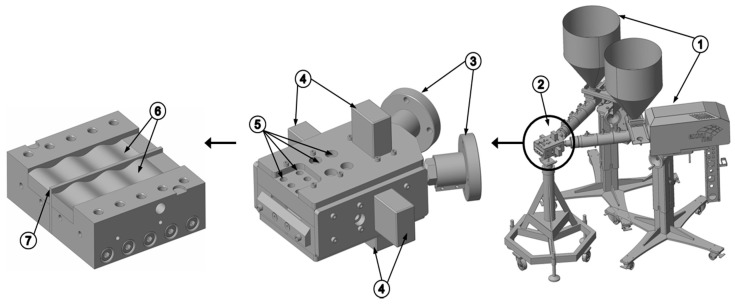
Components of the test device: (**1**) single-screw extruders; (**2**) extrusion die; (**3**) adaptors; (**4**) heating plates; (**5**) holes for measurement device; (**6**) sinusoidal flow channels; and (**7**) barrier flight.

Experimental studies were performed on a new extrusion die patented in Reference [21]. Reproducing the geometry of a dual wave-dispersion zone, this novel test apparatus incorporated two parallel flow channels with oscillating channel depth profiles that were separated by a barrier flight. A schematic of the assembly is shown in Figure 4.

Two ece-30 single-screw extruders (Extrunet, Kremsmuenster, Austria) equipped with a standard extruder screw of outer diameter *D*_b_ = 30 mm and axial length *L* = 606 mm (20.2·*D*_b_) were employed to feed the extrusion die with molten polymer. To heat the processing machines, thermal energy was supplied by electrical heaters grouped into four heating zones, including (i) three heating zones along the barrel, *T*_1_ to *T*_3_, and (ii) one heating zone for the adaptor *T*_4_ between the extruders and the die. The temperature of the die was adjusted by four heating plates grouped into two heating zones, *T*_5_ and *T*_6_. Table 3 shows the temperature profiles used in the experimental part.

**Figure 5:**
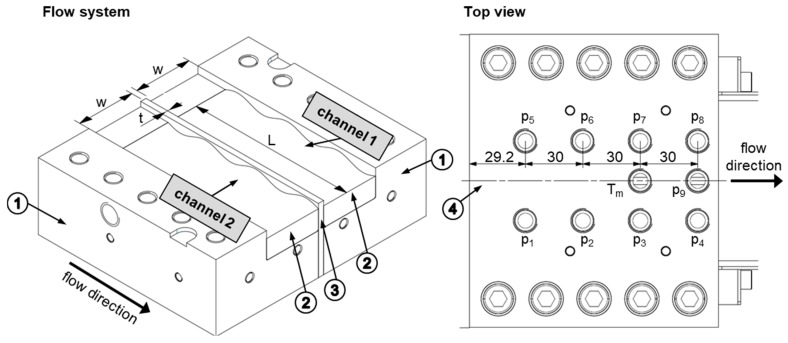
The left-hand side shows the main components of the extrusion die: (**1**) bottom plates, (**2**) flow channels, (**3**) barrier flight, and (**4**) top plate. The right-hand side illustrates a top view of the die, indicating the positions of the pressure transducers and the melt temperature sensor.

Figure 5 shows a schematic of the extruder die, including its main mechanical elements: (i) bottom plates, (ii) top plate, (iii) flow channels with alternating channel depth profiles, and (iv) a barrier flight that forms an undercut with the top plate. For convenience, the components were constructed as interchangeable elements. In this study, we investigated flow channels with alternating sinusoidal channel depth profiles. Table 4 lists the geometrical parameters of the wave channels. To promote cross-channel mixing, the phase shift between the channels was set to π/2. The following relationships were applied to describe the channel depth profiles mathematically:

(3) $$\mathrm{channel}\;1:\;h\left(z\right)=h_{v}-\left(a_{s}+a_{s}\cdot \sin\left(\frac{2\pi n}{L}z\right)\right)$$

(4) $$\mathrm{channel}\;2:\;h\left(z\right)=h_{v}-\left(a_{s}-a_{s}\cdot \sin\left(\frac{2\pi n}{L}z\right)\right)$$

where *h*_v_ is the channel depth in the valley.

Nine piezoresistive pressure transducers were placed in the top plate to measure the pressure distribution in the wave-dispersion zone. For each channel, four pressure sensors (p_1_ to p_4_ and p_5_ to p_8_) were located along the downchannel direction. The last pressure transducer, p_9_, was positioned over the top of the barrier flight at the end of the flow domain. Further, the temperature of the polymer melt was measured by means of a melt temperature sensor. Table 5 summarizes the technical properties of the pressure transducers. In addition, to measure the back pressures of the wave channels, two piezoresistive pressure sensors, p_01_ and p_02_, were placed in the adaptors.

**Figure 6:**
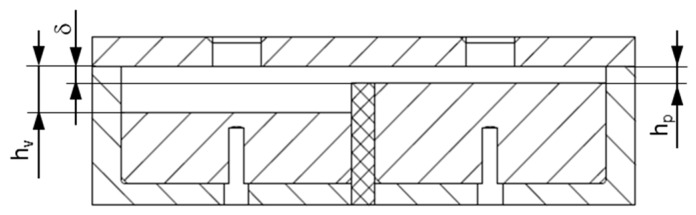
Cross-sectional view of the flow channels.

We compared four die assemblies by combining three top plates and two barrier flights (Table 6). One of the top plates was designed as a straight rectangular plate, whereas the other two were equipped with a small gap ranging into the flow domain. In this manner, both the channel depths along the wave channels and the undercut of the barrier flight were adjusted. Figure 6 represents a cross-sectional view of the flow channels. For the widest configuration, the maximum channel depth (at the wave valley) was 7.5 mm, and the minimum channel depth (at the wave peak) was 2.7 mm. For the narrowest setup, the maximum channel depth was 5.5 mm, and the minimum channel depth was 0.7 mm. The flight clearance varied from 0.7 to 2.7 mm. For all assemblies, the alternating sinusoidal form of the channel depth profiles, with three peaks and a valley, was kept uniform.

### 2.3. Procedure

Experiments were carried out by increasing the screw speed of the extruders synchronously from 10 to 150 rpm in equal steps of 20 rpm. It should be pointed out that, due to pressure limitations, we were not able to reach all screw speeds for each die assembly and material. With the processing machines operating at steady state, the total mass flow rate, the pressures in the die (*p*_1_ to *p*_9_) and in the adaptors (*p*_01_ and *p*_02_), and the melt temperature were measured. Considering the steady-state conditions of the process, we assumed that parameter fluctuations were low and that local changes in the measured values were negligible. Table A1 in Appendix A lists the set of processing conditions investigated in the experimental part. The performance data showed that even at low throughputs, the pressures in the adaptors deviated. This result was caused by the cross-channel flows in the wave-dispersion zone, which additionally affected the back pressures of the channels. A maximum difference of 18 bar was observed in the case of test 21 and 24.

Furthermore, to visualize cross-channel mixing, solidification experiments were carried out for selected processing conditions. To this end, the materials in the feeding systems were colored differently by using a masterbatch (4%). These trials required the single-screw extruders to be abruptly stopped and the heating zones of the die to be deactivated, thereby solidifying the polymer melt in the flow channels. After demounting the top plate, the solidified ribbon of polymer was extracted from the die.

**Figure 7:**
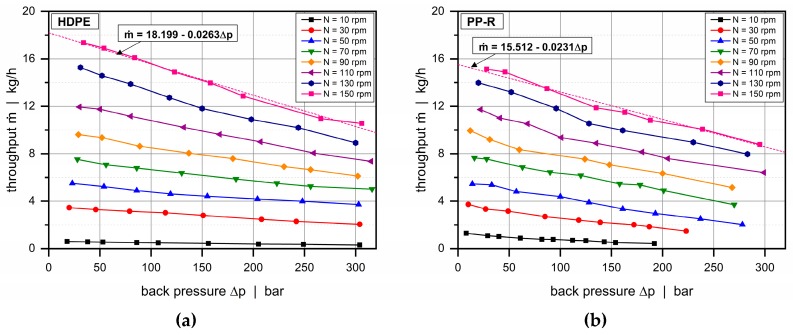
Screw characteristic curves for HPDE (**a**) and PP-R (**b**). A linear function was applied to describe the throughput–back pressure relationship for a screw speed of 150 rpm (mathematically).

Screw characteristic curves were evaluated to examine the pumping capability of the ece-30 single-screw extruder. Due to the cross-channel flows in the wave section, the back pressures of the flow channels deviated, causing the flow rates in each subchannel to be slightly different. To determine the pumping characteristics of the extruder, the processing machine was equipped with a valve at the discharge end, which allowed us to control the back pressure. For a constant screw speed, the back pressure was increased stepwise, and the mass flow rate was measured. Linear functions were used to describe the throughput–back pressure relationship mathematically, as shown in Figure 7.

## 3. Network Analysis

A seminumerical modeling approach based on network theory was developed and implemented in MATLAB to model the flows in the wave-dispersion zone. Our objective was to reproduce the pressure characteristics of the wave system by using the geometrical parameters of the flow domain, the material properties, and the processing conditions measured in the experimental part (e.g., mass flow rate and melt temperature) as input parameters.

**Figure 8:**
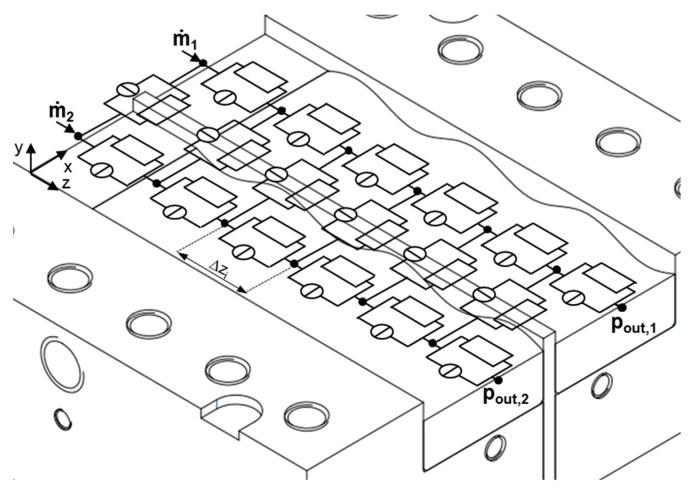
Flow network of a dual wave section, consisting of down- and cross-channel elements. The mass flow rates at the inlets are *ṁ*_1_ and *ṁ*_2_, and the pressures at the outlets are *p*_out,1_ and *p*_out,2_.

**Figure 9:**
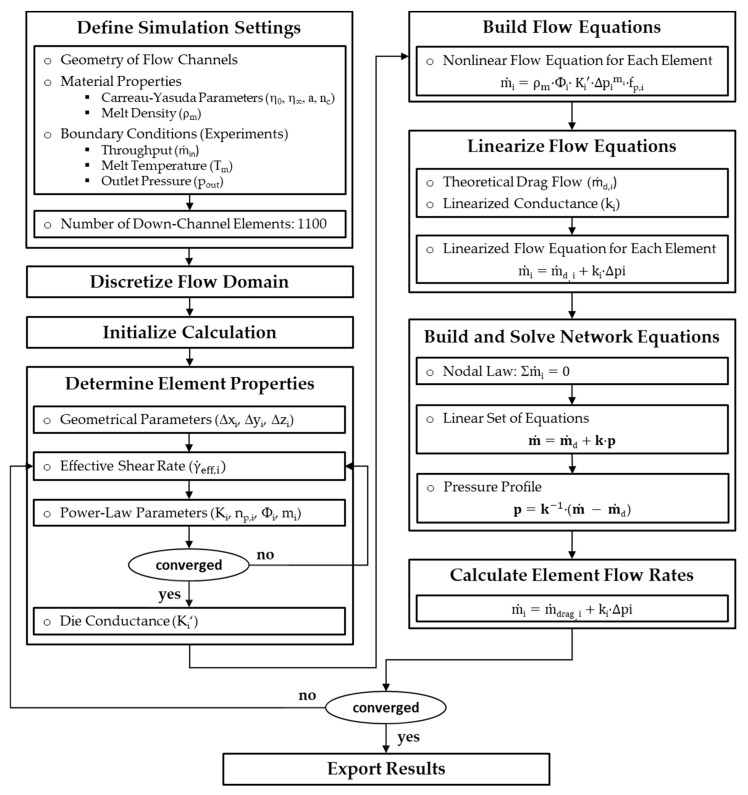
Flow chart of our seminumerical modeling approach based on network theory.

Network theory originates from the field of electrical engineering, where it is commonly used for calculating electrical networks based on Kirchhoff’s laws [22]. Two modeling approaches can be distinguished: (i) nodal and (ii) mesh analysis. Replacing the currents with flow rates, the voltages with pressures, and the electrical resistances with flow resistances, network theory has also proven useful in the field of polymer processing, where it has been successfully applied in modeling the flows in extrusion dies [23,24,25,26,27] and in extruders [28,29,30]. The main idea is to reduce the complexity of a multidimensional flow by subdividing the geometry into small passages for which simple analytical flow equations are available, assuming that both geometrical parameters and processing conditions are locally constant. Similarly to electrical circuits, these geometrically simpler sections are connected via nodal points to form an equivalent flow network, which is then solved in a manner analogous to nodal analysis or mesh analysis. For non-Newtonian fluids, an iterative procedure is additionally required to reach converging solutions. Figure 8 shows a schematic of the equivalent flow network for the wave-dispersion zone analyzed in this study. A flow chart of our seminumerical modeling approach is given in Figure 9.

We describe the shear-thinning flow behavior of the polymer melt by an Ostwald–deWaele power law model [31]:

(5) $$\eta_{p}=K\cdot {\dot{\gamma }}^{n_{p}-1}$$

where *K* is the consistency, and *n_p_* is the power law index. The fluidity *Φ* and the flow exponent *m* result from

(6) $$K=\frac{1}{\Phi^{1/m}}\;\mathrm{and}\;n_{p}=\frac{1}{m}$$

For power law fluids, the pressure-induced flow through a rectangular slit can be described by a nonlinear relationship [26]:

(7) $$\dot{m}=\rho_{m}\cdot K^{\prime }\cdot \Phi \cdot \Delta p^{m}\cdot f_{p}\;\mathrm{with}\;K^{\prime }=\frac{w\cdot h^{m+2}}{2^{m+1}\left(m+2\right)}{\left(\frac{1}{L}\right)}^{m}$$

This simple analytical equation relates the mass flow rate $\dot{m}$ and the pressure consumption Δ*p* via the melt density ρ_m_, the fluidity Φ, the flow exponent *m*, the die conductance *K*’, and a correction factor *f*_p_ (defined in Reference [27]). The latter is a function of the aspect ratio of the flow channel and takes the rate-limiting influence of the walls for a Newtonian fluid into account.

Our simulation routine was based on the following steps. At the beginning, basic simulation settings were defined. These included the geometry of flow channels, material properties, and boundary conditions such as inlet mass flow rates, outlet pressures, and melt temperature, which were measured in the experimental part. Considering an isothermal flow, the last of these was used to shift the viscosity data to the desired temperature level. Further, due to the incompressible nature of the polymer melt, the melt density of the materials was assumed to be constant. To this end, we used the densities measured at 200 °C and 200 bar (Table 2).

In the next step, the flow domain was discretized into a network of smaller segments of constant geometry. These geometrically simpler sections were represented by network elements, each of which consisted of a source and a resistance connected in parallel. The first indicated a local (theoretical) drag flow and the second the local pressure flow. Note that the drag flow component had no physical relevance but was required to transform the nonlinear flow equations into a linear form. Hence, the mass flow rate of an element is defined by the following linear superposition:

(8) $$\dot{m}={\dot{m}}_{d}+{\dot{m}}_{p}={\dot{m}}_{d}+k\cdot \left(p_{\mathrm{in}}-p_{\mathrm{out}}\right)$$

where ${\dot{m}}_{d}$ is the (theoretical) drag flow, ${\dot{m}}_{p}$ is the pressure flow, *k* is the linearized conductance, and *p*_in_ and *p*_out_ are the pressures at the surrounding nodal points.

**Figure 10:**
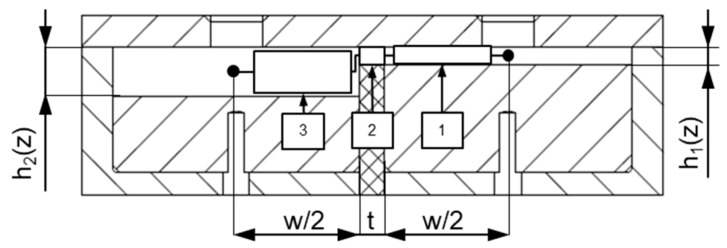
Initialization of the cross-channel connections over the barrier flight.

The resolution of the network was determined by the number of downchannel elements, which was set to *N_z_* = 1100 for all calculations, yielding a downchannel distance between adjacent nodes of Δ*z_i_* = 0.1 mm. Taking the reduced length of a network element into account, we applied the lubrication approximation [32], i.e., the flow was locally assumed to take place between two parallel plates. As a result, any motion of fluid in a direction normal to the surfaces could be neglected in comparison to a motion parallel to them. To include cross-channel flow, nodal points at the same downchannel position were connected in the direction perpendicular to the flight (Figure 10). These connections were initialized with three elements (with *h*_1_ = *h*_1_*(z)* and *w*_1_ = *w*/2, *h*_2_ = *δ* and *w*_2_ *= t*, *h*_3_ = *h_2_(z)* and *w*_3_ = *w*/2) and then replaced by one equivalent element in order to describe the stepwise changes in channel height in the cross-channel direction. The total drag flow and conductance of three elements connected in a series is given by

(9) $$\frac{1}{K_{\mathrm{total}}^{\prime }}={\left(\sum_{i=1}^{3}\frac{1}{K_{i}^{\prime }{}^{\frac{1}{m}}}\right)}^{m}\;\mathrm{and}\;{\dot{m}}_{d,\mathrm{total}}=\left(\sum_{i=1}^{3}\frac{{\dot{m}}_{d,i}}{K_{i}^{\prime }}\right)\cdot K_{\mathrm{total}}^{\prime }$$

The calculation was started by initializing the element properties, element flow rates, and nodal pressures with zeroes. In addition, the boundary conditions at the inlet and outlet of the flow domain were specified as follows: *ṁ*_1_ = *ṁ*_2_ = *ṁ*/2 and *p*_out,1_ = *p*_out,2_ = *p*_out_ = 0 bar (*ṁ* is the measured output). For convenience, the inlet mass flow rates in each subchannel were equally set to one-half of the total throughput. This assumption was not entirely correct. Taking the different back pressures in adaptors 1 and 2 into account, the flow rates in the subchannels will slightly deviate. When analyzing the screw characteristic curves in Figure 7, however, it can be seen that this difference was negligibly small.

**Figure 11:**
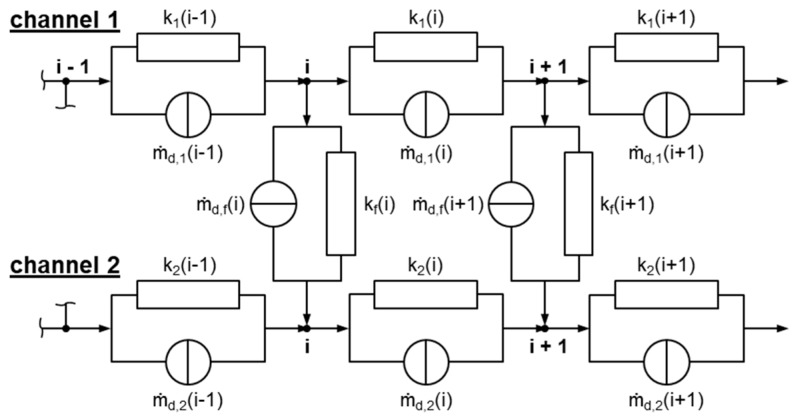
Equivalent circuit diagram.

In the final step, the equivalent circuit diagram (Figure 11) was solved. To this end, the network equations were built at each node by means of nodal analysis, assuming that the sum of the incoming flows (currents) must equal the sum of outgoing flows (cf. Kirchhoff′s current law):

(10) $$\sum_{i}{\dot{m}}_{i}=0$$

For an arbitrary nodal point with index *i*, the network equations are given by

(11) $$\begin{array}{cc} \mathrm{channel}\;1: & k_{1}\left(i-1\right)p_{1}\left(i-1\right)+\left[-k_{1}\left(i-1\right)-k_{1}\left(i\right)-k_{f}\left(i\right)\right]p_{1}\left(i\right)+k_{f}\left(i\right)p_{2}\left(i\right)+\\ & k_{1}\left(i\right)p_{1}\left(i+1\right)=-{\dot{m}}_{d,1}\left(i-1\right)+{\dot{m}}_{d,1}\left(i\right)+{\dot{m}}_{d,f}\left(i\right) \end{array}$$

(12) $$\begin{array}{cc} \mathrm{channel}\;2: & k_{2}\left(i-1\right)p_{2}\left(i-1\right)+k_{f}\left(i\right)p_{1}\left(i\right)+\left[-k_{2}\left(i-1\right)-k_{2}\left(i\right)-k_{f}\left(i\right)\right]p_{2}\left(i\right)+\\ & k_{2}\left(i\right)p_{2}\left(i+1\right)=-{\dot{m}}_{d,2}\left(i-1\right)+{\dot{m}}_{d,2}\left(i\right)-{\dot{m}}_{d,f}\left(i\right) \end{array}$$

Taking all nodes of the flow into account, a linear system of equations can be set up in matrix form:

(13) $$\dot{m}={\dot{m}}_{d}+k\cdot p$$

where *ṁ*_d_ is the drag flow vector, *k* is the linearized conductance matrix, *p* is the pressure vector, and *ṁ* includes the boundary conditions. The pressure field of the flow domain is hence obtained from

(14) $$p=k^{-1}\left(\dot{m}-{\dot{m}}_{d}\right)$$

Special attention has to be placed on the development of a network equation for the first node, where the inlet mass flow rates are known:

(15) $$\mathrm{channel}\;1:\;\left[-k_{1}\left(1\right)-k_{f}\left(1\right)\right]p_{1}\left(1\right)+k_{f}\left(1\right)p_{2}\left(1\right)+k_{1}\left(1\right)p_{1}\left(2\right)=-{\dot{m}}_{1}+{\dot{m}}_{d,1}\left(1\right)+{\dot{m}}_{d,f}\left(1\right)$$

(16) $$\mathrm{channel}\;2:\;k_{f}\left(1\right)p_{1}\left(1\right)+\left[-k_{2}\left(1\right)-k_{f}\left(1\right)\right]p_{2}\left(1\right)+k_{2}\left(1\right)p_{2}\left(2\right)=-{\dot{m}}_{2}+{\dot{m}}_{d,2}\left(1\right)-{\dot{m}}_{d,f}\left(1\right)$$

Similarly, the equations must be modified for the last nodes, where the outlet pressures are predefined:

(17) $$\begin{array}{cc} \mathrm{channel}\;1: & k_{1}\left(n-1\right)p_{1}\left(n-1\right)+\left[-k_{1}\left(n-1\right)-k_{1}\left(n\right)-k_{f}\left(n\right)\right]p_{1}\left(n\right)+k_{f}\left(n\right)p_{2}\left(n\right)=\\ & -k_{1}\left(n\right)p_{\mathrm{out},1}-{\dot{m}}_{d,1}\left(n-1\right)+{\dot{m}}_{d,1}\left(n\right)+{\dot{m}}_{d,f}\left(n\right) \end{array}$$

(18) $$\begin{array}{cc} \mathrm{channel}\;2: & k_{2}\left(n-1\right)p_{2}\left(n-1\right)+k_{f}\left(n\right)p_{1}\left(n\right)+\left[-k_{2}\left(n-1\right)-k_{2}\left(n\right)-k_{f}\left(n\right)\right]p_{2}\left(n\right)=\\ & -k_{2}\left(n\right)p_{\mathrm{out},2}-{\dot{m}}_{d,2}\left(n-1\right)+{\dot{m}}_{d,2}\left(n\right)-{\dot{m}}_{d,f}\left(n\right) \end{array}$$

Solving the network equation (Equation (14)) requires the properties of the network elements to be evaluated. The dimensions of the elements are determined by using the geometrical parameters known at each nodal point. These parameters are applied to calculate the local correction factors in the die equation (Equation (7)). Next, the local shear rate is evaluated for each element:

(19) $${\dot{\gamma }}_{\mathrm{eff},i}=\frac{6{\dot{m}}_{i}}{\rho_{m}w_{i}h_{i}^{2}}{\left(\frac{3n_{p,i}}{2n_{p,i}+1}\right)}^{\frac{n_{p,i}}{1-n_{p,i}}}$$

**Figure 12:**
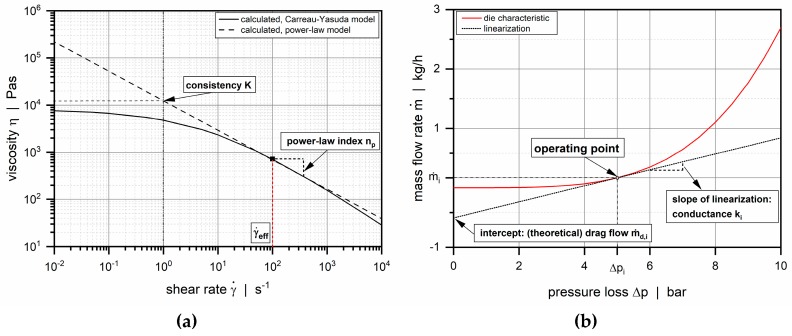
Conversion of Carreau–Yasuda parameters into power law parameters (**a**) and linearization to the die characteristic curve at operating point (Δ*p_i_* | *ṁ*_i_) (**b**).

Since the local power law parameters are initially unknown, this step is based on an iterative procedure. On a log–log scale, the power law can be considered as the tangent of the Carreau–Yasuda model at a specific shear rate, as shown in Figure 12a. The local power law parameters are obtained from the slope and the intercept of the tangent:

(20) $$n_{p}=\frac{\left(\eta_{0}-\eta_{\infty }\right)\left(n_{c}-1\right){\left(a_{t}\lambda {\dot{\gamma }}_{\mathrm{eff},i}\right)}^{a}{\left(1+{\left(a_{t}\lambda {\dot{\gamma }}_{\mathrm{eff},i}\right)}^{a}\right)}^{\frac{n_{c}-1-a}{a}}}{\eta_{\infty }+\left(\eta_{0}-\eta_{\infty }\right){\left(1+{\left(a_{t}\lambda {\dot{\gamma }}_{\mathrm{eff},i}\right)}^{a}\right)}^{\frac{n_{c}-1-a}{a}}}$$

(21) $$K=\eta_{\infty }+\left(\eta_{0}-\eta_{\infty }\right){\left(1+{\left(a_{t}\lambda {\dot{\gamma }}_{\mathrm{eff},i}\right)}^{a}\right)}^{\frac{n_{c}-1}{a}}{\dot{\gamma }}_{\mathrm{eff},i}^{1-n_{p,i}}$$

The power law parameters are then used to determine the die conductance in Equation (7), which is linearized at the local operating point (Figure 12b). For each element, (theoretical) drag flow and conductance are obtained from the initial value and the slope of the linearization. At the end of the procedure, the linear set of network equations is solved, and the calculated pressure field is used to update the element flow rates for the next iteration. A simulation was considered converged if the pressure differences between the first and the final nodes in each channel were smaller than 0.01 bar.

A special feature of the seminumerical procedure presented here is the linearization applied to build the network equations at each nodal point. Previous studies dealing with flow resistance networks [25,27] have used the concept of representative viscosities [33,34] to include shear-thinning flow behavior of the polymer melt. By linearizing the nonlinear flow equation (Equation (7)) for each network element, our approach inherently considers shear-thinning flow behavior.

## 4. CFD Simulation

Three-dimensional numerical flow simulations were carried out using the software package ANSYS Fluent, which is based on the finite volume method. The main objective was to compare the results of our seminumerical modeling approach to the solutions of a widely known numerical technique.

**Figure 13:**
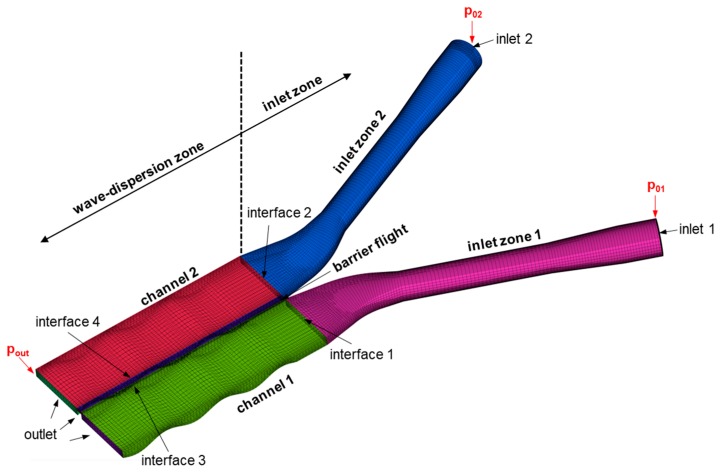
Schematic of the computational domain. Interfaces were used to merge the meshes of each subsection.

In this numerical analysis, the fluid domain was extended to include the flow channels formed by the adaptors. Five subsections were defined: inlet zones 1 and 2, channels 1 and 2, and the barrier flight. Each of these cell zones was meshed separately and then merged via interfaces to obtain the computational domain, as shown in Figure 13. For all subsections, hexahedral elements were used. In total, 124,332 hexahedral elements were employed.

Considering a stationary flow of an incompressible fluid, the conservation equations of mass and momentum given in Reference [35] were reduced to

(22) ∇·(v)=0

(23) $$\nabla \cdot \left(\rho_{m}vv\right)=-\nabla p+\nabla \cdot \tau$$

where ρ_m_ is the melt density, *v* is the velocity vector, *p* is the pressure, and *τ* is the stress tensor. Since an isothermal flow was considered, the energy equation was omitted. The following relationship was applied to express the constitutive nature of the polymer melt:

(24) $$\tau =2\eta_{c}\left(\dot{\gamma }\right)D\;\mathrm{with}\;D=\frac{1}{2}\left(L+L^{T}\right)\;\mathrm{and}\;L=\nabla v$$

where η_c_ is the melt viscosity defined by the Carreau–Yasuda model in Equation (1) and the material properties in Table 2. The rate of deformation tensor *D* is given by the symmetric part of the velocity gradient tensor *L*. To simulate the flows in the wave zone for selected processing conditions, the adaptor pressures p_01_ and p_02_ were predefined according to the experimental results (Table A1), whereas the pressure at the outlet was fixed to p_out_ = 0 bar. Assuming a wall-adhering polymer melt, the fluid velocities at the walls of the flow domain were set to zero.

To solve the flow equations, spatial discretization was carried out by means of second-order upwind functions, and pressure–velocity coupling was solved using the SIMPLE algorithm (Semi-Implicit Method for Pressure Linked Equations) [36]. The iteration number was fixed such that both numerical convergence (residuals) and physical convergence (volume flow rate at the outlet surface) were reached. For each simulation, the total mass flow rate and the pressure profiles along the wave channels were evaluated. Streamlines in both wave channels were colored differently and then tracked along their flow paths to visualize cross-channel mixing.

## 5. Results and Discussion

### 5.1. Experiments

#### 5.1.1. Influence of Channel Depth

**Figure 14:**
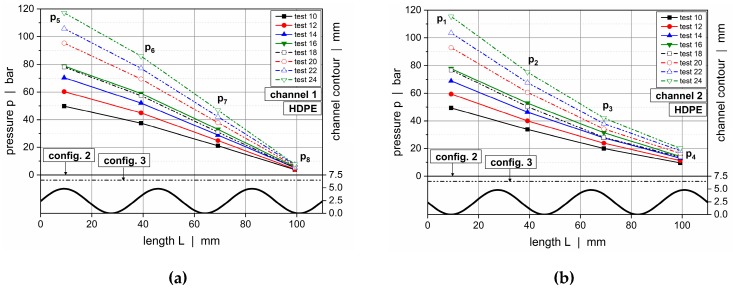

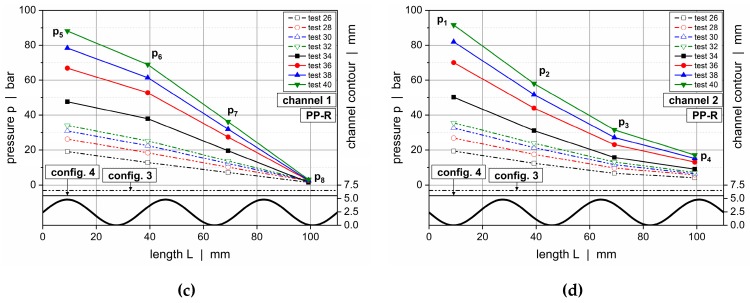
Downchannel pressure profiles along channel 1 and 2 for various test configurations: channel 1, HDPE, configuration 2 versus configuration 3 (**a**); channel 2, HDPE, configuration 2 versus configuration 3 (**b**); channel 1, PP-R, configuration 3 versus configuration 4 (**c**); and channel 2, PP-R, configuration 3 versus configuration 4 (**d**).

The influence of the channel depth on the pressure characteristics of the wave system is demonstrated in Figure 14, which compares downchannel pressure profiles along channels 1 and 2 for HDPE (setup 2 vs setup 3) and PP-R (setup 3 vs setup 4).

Configuration 2 showed a maximum channel depth of 7.5 mm and a minimum of 2.7 mm. With the top plates of the latter setups ranging into the flow domain, the channel depths of configurations 3 and 4 were collectively reduced by 1 and 2 mm, respectively. For all setups, the undercut of the barrier flight was adjusted to guarantee a plane transition between the wave peaks and flight land (Figure 6). For convenience, the contours of the wave channels were indicated using thick continuous lines. The straight lines above represent the positions of the top plates compared in each case, whereby the dashed and continuous lines are associated with the dashed and continuous pressure curves, respectively.

For all operating conditions, similar pressure characteristics were evident. The pressure gradient increased while the molten resin flowed toward a peak, and it decreased while the polymer melt approached a valley. Since the channel depth profiles oscillated out of phase, these unbalanced pressure conditions forced the material to flow across the barrier flight in the direction of the channel where the pressure was locally reduced. Since the material transfer over the secondary flight was considered, the pressure gradient before a peak was expected to be larger than after a peak given the reduced local flow rate in the latter case. Consequently, the local pressure characteristics were not only affected by the geometrical flow resistances of the channels but also by the cross-channel flow over the barrier flight. The largest pressure gradients were found in the vicinity of a peak, whereas the lowest gradients occurred in the region of a valley. Comparing the influence of the geometrical configurations, the downchannel pressure gradient increased the lower the channel depths of the system were.

**Figure 15:**
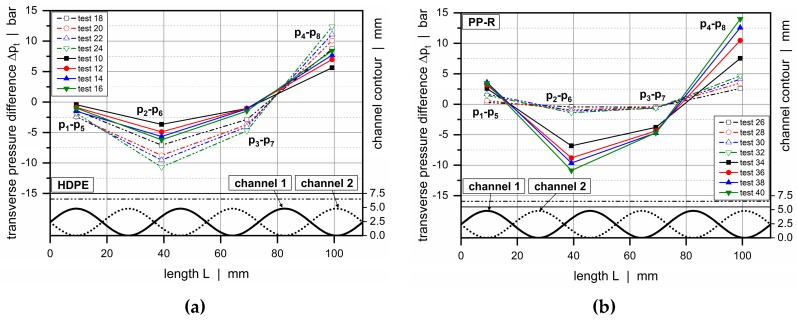
Transverse pressure difference between channel 2 and channel 1 for various test configurations: HDPE (a) and PP-R (b).

A closer look at the transverse pressure difference between the subchannels is taken in Figure 15. This parameter provides an important measure in the analysis of cross-channel mixing, since it determines the level of material transfer over the secondary flight. The cross-channel pressure difference changed its sign and magnitude along the downchannel direction. A pronounced negative value, on the one hand, was obvious after roughly 40 mm. In this region, the channel depth of channel 1 decreased while the other increased, causing a material transfer from the first into the second subchannel. A pronounced positive value, on the other hand, was found after 100 mm. Here, the channel depth in channel 1 was at a maximum, whereas the other was at a minimum, forcing the material to flow in the opposite direction. A reduction in channel depths increased the transverse pressure gradients and hence promoted cross-channel mixing.

#### 5.1.2. Influence of Viscosity Behavior

**Figure 16:**
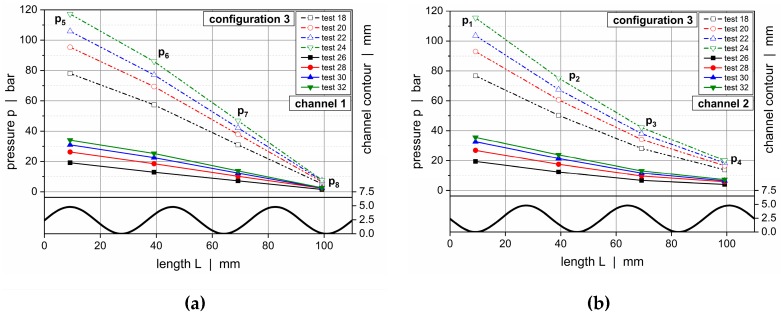
Down-channel pressure profiles along channel 1 (**a**) and channel 2 (**b**) for HDPE and PP-R for configuration 3.

**Figure 17:**
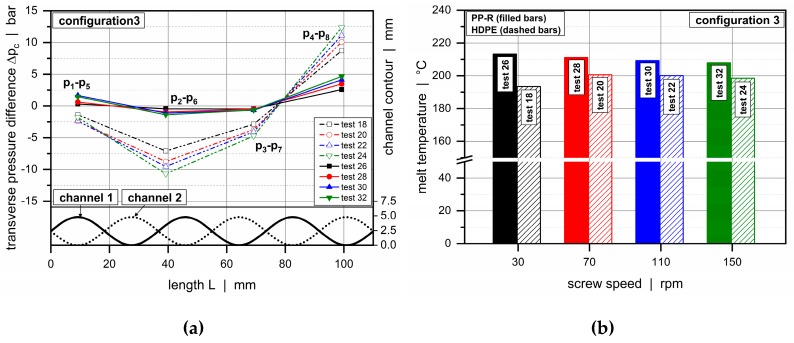
Influence of viscosity behavior. Transverse pressure difference between channel 2 and channel 1 (**a**) and melt temperatures (**b**) for configuration 3.

The influence of the viscosity behavior of the polymer melt on the pressure characteristics of the wave system is demonstrated in Figure 16 and Figure 17a for configuration 3. This assembly shows a maximum channel depth of 6.5 mm and a minimum of 1.7 mm. Again, the undercut of the barrier flight was adjusted to avoid gaps between the wave peaks and flight land.

The rheological behavior of the molten resin exhibited a distinct impact on the conveying characteristics. The higher the viscosity of the polymer melt, the more pronounced was the pressure consumption at the same throughput. Referring to the thermal behavior of the materials (Figure 17b), the melt temperature of the highly viscous HDPE rose with increasing throughput, whereas the lower viscous PP-R decreased. This result was related to the change in specific energy input, i.e., the frictional heat generation per unit throughput. For both materials, viscous dissipation rose with an increasing output rate. In the case of HDPE, this change prevailed over the increase in throughput. As a result, the energy dissipation per unit throughput went up. In the case of PP-R, in contrast, the change in throughput exceeded the additional frictional heat generation, and the energy dissipation per unit throughput decreased, leading to a lower melt temperature. The change in specific energy input may also have been a result of the varying flow conditions in the plasticating units.

#### 5.1.3. Influence of Flight Clearance

**Figure 18:**
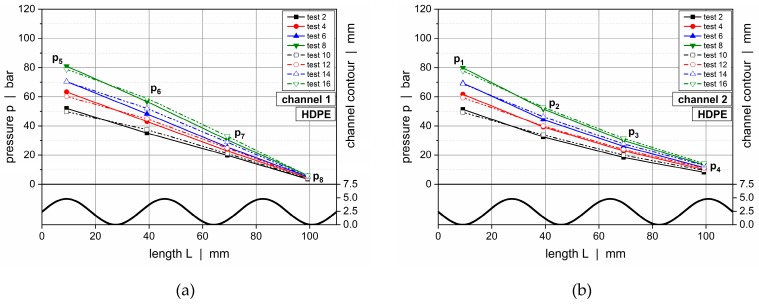
Comparison of axial pressure profiles along channel 1 (**a**) and channel 2 (**b**) for test configurations 1 and 2.

The influence of the flight clearance on the die characteristics for HDPE and test configurations 1 and 2 is illustrated in Figure 18. These setups, which were almost identical from a geometrical viewpoint, showed a maximum channel depth of 7.5 mm and a minimum of 2.7 mm. Since the magnitude of the flight clearance was different for both assemblies, the undercut of the barrier flight was 1.7 mm for setup 1, whereas the flight clearance was 2.7 mm for setup 2.

The undercut of the barrier flight showed a diminished influence on the pressure characteristics. Constructing the barrier flight with a smaller undercut caused the wave-dispersion zone to consume slightly more pressure. Depending on the exact downchannel position, however, a larger undercut could locally cause increased pressures, since transverse flow became more pronounced (Figure 18). Note that if the undercut of the barrier flight was too small, transverse flow and thus cross-channel mixing was almost totally restricted.

### 5.2. Comparison between Experiment, Network Calculation, and CFD Simulation

#### 5.2.1. Downchannel Pressure Profiles

**Figure 19:**
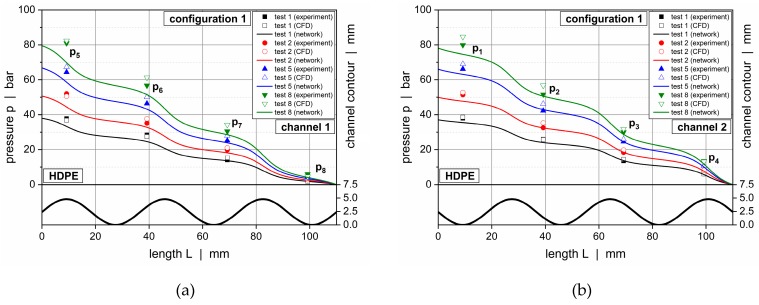
Comparison between experimental data and calculated solutions (seminumerical approach based on network theory vs CFD simulation) for selected operating conditions. Downchannel pressure profiles along channel 1 (**a**) and channel 2 (**b**) for HDPE and configuration 1.

**Figure 20:**
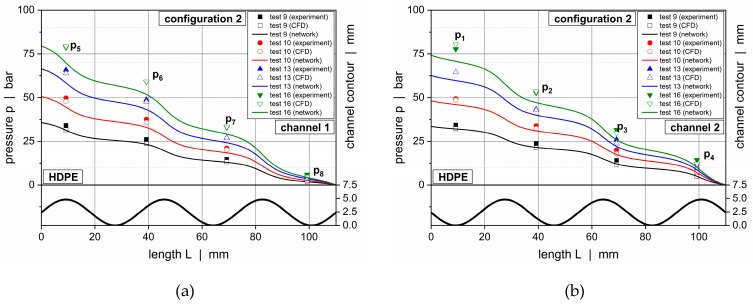
Comparison between experimental data and calculated solutions (seminumerical approach based on network theory vs CFD simulation) for selected operating conditions. Downchannel pressure profiles along channel 1 (**a**) and channel 2 (**b**) for HDPE and configuration 2.

Figure 19 and Figure 20 illustrate downchannel pressure profiles for HPDE and configurations 1 and 2. The solutions according to our seminumerical modeling approach are represented by continuous lines. The empty symbols indicate numerical results, whereas the filled symbols show the measured data. Each diagram provides a comparison of four throughputs.

The pressure profiles obtained from the seminumerical modeling approach were in good agreement with the measured data. This was particularly true if the throughput was low. Minor deviations were observed at the position of the first pressure transducer (*p*_1_ and *p*_5_), where differences in the range of 10% were evident. In contrast to the experimental data, the calculated curves provided a full description of the pressure behavior over the entire length of the wave channels, thus allowing a local assessment of the pressure characteristics at each position of the channels. As expected, the largest downchannel pressure gradients were found in the region of the wave peaks, whereas the lowest appeared in the vicinity of the valleys.

Comparing the solutions of both the seminumerical and numerical methods, a slightly higher accuracy was given in the case of the three-dimensional CFD simulations. This result was mainly caused by two factors. First, the numerical approach was based on a finer discretization of the computational domain, hence allowing for a more accurate representation of the local flow mechanisms. Second, rather than linearizing an analytical equation for pressure-induced flows, the numerical procedure solved the full set of conservation equations for each cell. Taking the increased time for the numerical solving procedure into account, however, the results of the seminumerical approach provided a useful approximation of the pressure characteristics.

**Figure 21:**
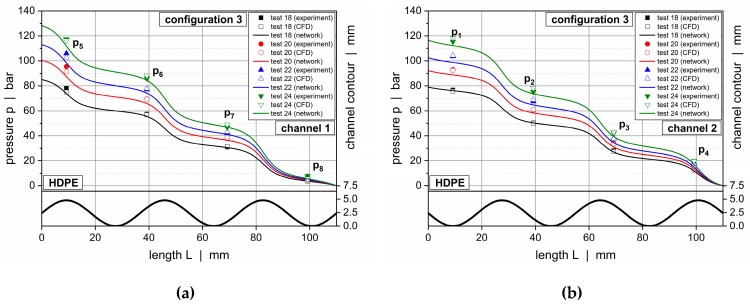

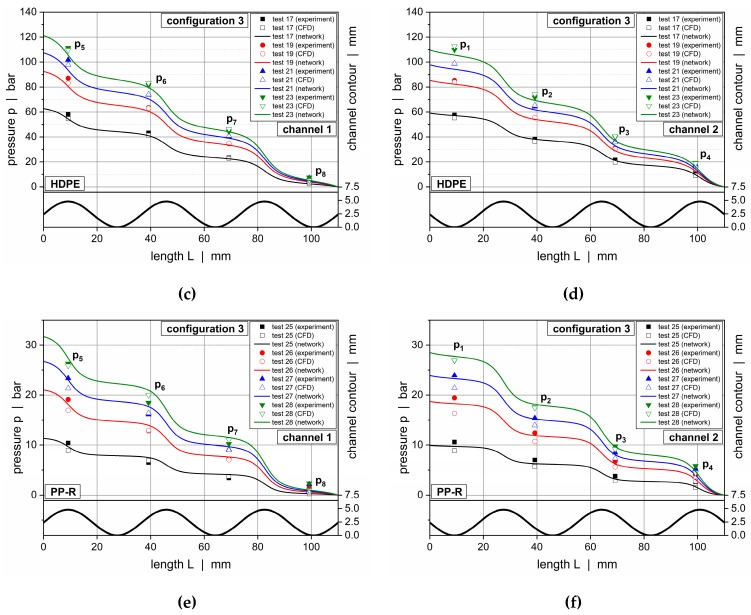
Comparison between experimental data and calculated solutions (seminumerical approach based on network theory vs CFD simulation). Downchannel pressure profiles for test configuration 3 and various materials: HDPE, channel 1 (**a**); HDPE, channel 2 (**b**); HDPE, channel 1 (**c**); HDPE, channel 2 (**d**); PP-R, channel 1 (**e**); and PP-R, channel 2 (**f**).

The deviations between the experimental and calculated results almost disappeared if the channel depths were reduced, as demonstrated in Figure 21 for configuration 3. In this case, the pressure profiles obtained from our seminumerical procedure were in excellent agreement with the experimental data for both materials. A reason for the improved accuracy was the correction factor in the nonlinear die (Equation (7)), which was based on a Newtonian fluid. With the polymer melt actually being shear-thinning, the calculation errors were reduced the shallower the flow channel was. Note that there was almost no difference in accuracy between the seminumerical and numerical solutions.

Considering the reduced complexity of the seminumerical analysis, its high accuracy was surprising, as the validity of a few modeling assumptions needed to be critically readdressed. A closer look had to be taken at the simplifications made in the derivation of the flow equation (Equation (7)). In its development, the flow was assumed to take place between two parallel plates, and therefore acceleration terms in the momentum equation were omitted, i.e., downchannel velocity gradients were ignored. Taking the alternating channel depth profiles of the wave-dispersion zone into account, this assumption may not have held, as even in the simplified network, the downchannel velocity was a function of the downchannel coordinate. It should be pointed out, however, that this influence was limited (d*v_z_*/d*z* ≪ d*v_z_*/d*y*). Another drawback came from the description of the flows over the barrier flight. By connecting nodal points in the direction perpendicular to the downchannel direction, the cross-channel elements were only capable of representing exactly these types of flows, i.e., the downchannel velocity component of the fluid over the flight land was ignored. Moreover, when calculating the effective shear rate for each network element, cross-channel flow was omitted. A more accurate assessment of the shear rates would require a consideration of flow components in both the cross-channel and downchannel direction. In addition, the procedure considered the polymer melt to be incompressible, assuming the density of the fluid to be constant. In fact, the density is a function of temperature and pressure. With the pressure being known at each nodal point of the flow network, the dependency of density on pressure could be included easily.

Our main objective was to strike a reasonable balance between the degree of accuracy and level of sophistication. When increasing the complexity of the analysis, a significant improvement in the results was not expected. In addition to the high accuracy of the seminumerical approach, its major advantage was the reduced calculation time. Using the same computer settings, the solving process was at least 15 times faster than solving the flow equation numerically by means of CFD simulations. This difference was additionally increased by orders of magnitude if the geometry needed to be varied. In this case, the seminumerical procedure required only a modification of input parameters, whereas for the numerical method a completely new computational domain must be designed.

#### 5.2.2. Cross-Channel Mixing

**Figure 22:**
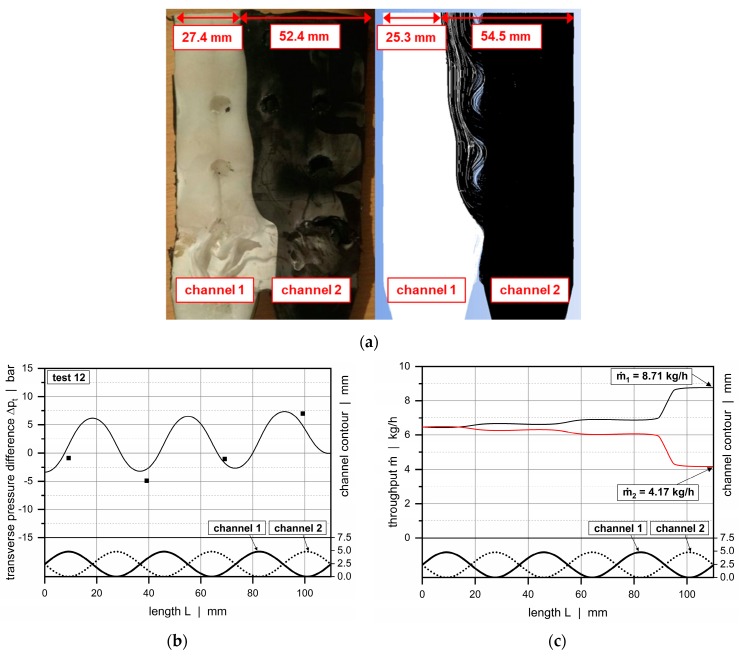
Comparison between experimental data and calculated solutions (seminumerical approach based on network theory vs CFD simulation). Analysis of cross-channel flow between the channels. Figure (**a**) shows a comparison of the experimental and numerically evaluated cross-channel flows. Figures (**b**,**c**) compare the calculated transverse pressure differences and throughputs according to our semi-numerical approach and CFD simulations.

Figure 22 analyzes the cross-channel flow behavior for operating point 12. For this selected test, HDPE was processed at a total throughput of 12.9 kg/h using die assembly 2. To visualize cross-channel mixing, a solidification experiment was carried out. Figure 22a illustrates a top view of solidified polymer ribbon extracted from the wave-dispersion zone, indicating the transverse flow of the polymer melt. The corresponding simulation result is illustrated.

After entering the dual wave section, white material flowed across the barrier flight from channel 1 into channel 2. Obviously, at this position, channel 1 was equipped with a wave peak, whereas channel 2 showed a valley: hence, the pressure in channel 1 was locally higher. A significant change was evident when channel 2 reached its first minimum channel depth and the black material was forced across the barrier flight due to the changing sign of the transverse pressure gradient. In contrast to the experimental sample, the simulation result clearly showed that mixing between white and black trajectories continued along the downchannel direction. This effect was not as clearly visible in the experimental sample, since a mixture of black and white was still black. For both analyses, however, the black material covered more space in channel 1 than vice versa until the end of the wave zone, demonstrating an increased material transfer from channel 2 into 1. This result was interesting, as both flow channels showed the same geometrical resistance, and the flow rates at the channel inlets were almost equal. We conclude that the overall cross-channel flow was mostly governed by the position of the final peak, which was located in channel 2.

The numerically evaluated flow field accurately described the experimental behavior. Relating the width of the black material segment at the end of the wave section to the total channel width of the system of 79.8 mm yielded a ratio of 0.66 and 0.68 for the experimental and numerical results, respectively. Similarly to the prediction of the downchannel pressure profiles, the seminumerical modeling approach was capable of reproducing the real physical behavior, as shown in Figure 22b,c. The first diagram presents a comparison between measured and calculated transverse pressure differences, in which the calculated continuous line accurately matches the measured values. The second diagram plots the mass flow rates of each subchannel as a function of the downchannel length, in which the oscillating curves indicate the material transfer across the barrier flight. Similarly, relating the increased throughput in channel 1 to the total output of 12.9 kg/h yielded a ratio of 0.68. These results show that the seminumerical method both qualitatively and quantitatively replicated the measured behavior.

## 6. Conclusions

Modeling the flow of polymer melts in wave-dispersion screws is a complex task. Due to the oscillating channel depth profile of the screw channel, the shear rate changes at each position, thereby locally affecting the viscosity and therefore the drag and the pressure flows in the downchannel and in the cross-channel directions. All of these components are coupled as a result of the shear-thinning flow behavior of the polymer melt. Although mathematical representations of the physical process can be derived, analytical solutions become elusive and in general time-consuming, and computational expensive numerical CFD simulations are required to solve the flow equations.

To remove the need for the latter, we propose an alternative seminumerical modeling approach, which enables a fast and accurate analysis of the flow phenomena. The main idea of the approach is as follows: By means of network theory, the flow domain is subdivided into very small passages of constant geometry, for which analytical equations are used. These smaller sections are connected via nodal points to form an equivalent flow network, which is solved using Kirchhoff’s law, i.e., for each nodal point the sum of incoming flow rates must equal the sum of outgoing flow rates. As a special feature of the modeling approach, a linearization method is applied to evaluate the properties of the network elements. A major advantage of this technique is that the flow can be locally described by the nonlinear flow equation (Equation (7)). This step increases the accuracy of the results, as the underlying flow equations inherently consider shear-thinning flow behavior. Another novel feature of the approach is the representation of transverse flows over the barrier flight. With the flow network in the cross-channel direction being initialized with three network elements connected in a series, the stepwise changes in channel height between the subchannels can be accurately described. For dual wave sections, these changes are significant when one subchannel reaches its valley and the other subchannel its peak. In this case, the channel depth in one subchannel is at a maximum and at a minimum in the other. The modeling approach presented here provides a convenient method for capturing the change in channel depth in the transverse direction.

The usefulness of the seminumerical modeling approach is the substantial reduction of calculation time compared to three-dimensional non-Newtonian CFD analyses. Rather than solving the full set of conservation equations, the seminumerical modeling approach iteratively solves a linearized set of network equations. Using the same computer settings, the latter is considerably faster, while it still provides satisfactory solutions. This effect is even more pronounced if an optimization study is carried out and the geometrical configuration of the wave section changes. This requires the creation of a new computational domain in the case of a CFD analysis and a modification of input parameters for our modeling approach. For various operating points, we showed that the results of the seminumerical modeling approach were nearly as accurate as the solutions of three-dimensional CFD simulations. Minor deviations could be explained by the diverse complexity of the mathematical model solved in both cases.

The validity of the seminumerical modeling approach was experimentally confirmed by comparing the calculated downchannel pressure profiles along the wave channels to measured data. For a variety of experimental setups, the solutions were in very good agreement with the measured data. In addition, the modeling approach was demonstrated to accurately predict the cross-channel flows along the wave zone by means of solidification experiments. As expected, the transverse mixing of polymer melt between the subchannels was limited, since the drag force of the rotating screw was omitted in this analysis. By simplifying the real physical process in single-screw extruders, however, we systematically reduced the complexity of the problem, which allowed us to validate our novel modeling approach and to clearly assign the results to the governing type of flow mechanism. Implementing the influence of screw rotation in the calculation did not substantially change the groundwork of the theory, as the approach already contained a drag flow variable in the network calculation. This component was needed for linearizing the nonlinear flow equations in the present study, whereas it provided the interface for including the actual physical drag flow in the analysis of extruder screws. In the latter case, the flow equation (Equation (7)) would be replaced by the two- and three-dimensional melt-conveying models developed in References [37,38,39,40,41]. Moreover, when analyzing wave-dispersion screws, leakage flow over the main flight has to be considered, which requires an extension of the flow network.

Our new seminumerical modeling approach enables a fast and stable prediction of the flows and the pressure demands of wave systems. The routine can therefore be used to quickly develop more effective geometrical designs. Apart from wave-dispersion zones, the method can be applied to model the flow in various types of extrusion dies, including flow channels with changing channel geometry. The analysis presented here will be extended to include the influence of the screw rotation on the flow behavior in Part B.

## Figures and Tables

**Table 1:** Comparison of the materials. HDPE: high-density polyethylene; PP-R: polypropylene random copolymer; MFR: melt flow rate.

Material	Type	MFR (ISO 1133)		Application	Manufacturer
HE3493-LSH	HDPE	0.24 g/10 min	(190 °C, 5 kg)	pipes	Borealis
RD204CF	PP-R	8.00 g/10 min	(230 °C, 2.16 kg)	films	Borealis

**Table 2:** Material properties (*ρ_m_* is the melt density at 200 °C and 200 bar).

Parameter	Unit	HDPE	PP-R
η_0_	Pas	95,230	2957
η_∞_	Pas	0	0
λ	s	3.256	0.173
*n*	-	0.289	0.425
*a*	-	1	1
α	1/K	0.0138	0.0162
*T*_0_	K	473.15	473.15
*ρ_m_*	kg/m^3^	752	730

**Table 3:** Temperature profiles in °C.

Material	*T*_1_	*T*_2_	*T*_3_	*T*_4_	*T*_5_	*T*_6_
HDPE	195	220	200	200	200	200
PP-R	195	230	220	220	220	220

**Table 4:** Geometrical parameters of the flow channels.

Dimensions	Parameter	Unit	Value
channel width	*w*	mm	38
flight width	*t*	mm	3.8
channel length	*L*	mm	110
number of waves	*n*	-	3
channel depth	*h*	mm	variable
channel depth (wave valley)	*h*_v_	mm	variable
channel depth (wave peak)	*h*_p_	mm	variable
flight clearance	*δ*	-	variable
amplitude of sine	*a_s_*	mm	2.4

**Table 5:** Positions and technical properties of pressure transducers. FSO: full scale output.

Properties	Unit	p_01_ and p_02_	p_1_	p_2_ to p_4_	p_5_	p_6_ to p_8_	p_9_
range	bar	0–1000	0–500	0–350	0–500	0–350	0–350
output signal	mA	4–20	4–20	4–20	4–20	4–20	4–20
response time (10%–90% FSO)	ms	8	8	8	8	8	8
position	-	Adaptors	Channel 2		Channel 1		Flight

**Table 6:** Set of test configurations.

Configuration	Top Plate	Flight	δ	*h*_p_	*h*_v_
-	No.	No.	mm	mm	mm
1	1	1	1.7	2.7	7.5
2		2	2.7		
3	2	2	1.7	1.7	6.5
4	3	2	0.7	0.7	5.5

## Appendix A

**Table A1:** Operating points.

Test	Configuration	Material	*N*	*ṁ*	*T*_M_	*p*_01_	*p*_02_
-	-	-	rpm	kg/h	°C	bar	bar
1	1	HDPE	10	2.6	189.1	67	65
2	1	HDPE	30	6.6	190.9	94	91
3	1	HDPE	50	9.8	192.3	107	104
4	1	HDPE	70	12.8	193.6	116	111
5	1	HDPE	90	16.9	192.5	127	120
6	1	HDPE	110	19.7	195.2	131	125
7	1	HDPE	130	25.4	194.9	145	140
8	1	HDPE	150	30.7	193.3	156	148
9	2	HDPE	10	2.3	196.8	59	55
10	2	HDPE	30	6.4	192.2	92	86
11	2	HDPE	50	9.7	192.0	106	98
12	2	HDPE	70	12.9	195.8	113	106
13	2	HDPE	90	17.2	198.3	124	115
14	2	HDPE	110	20.7	198.8	133	124
15	2	HDPE	130	25.1	196.8	143	135
16	2	HDPE	150	29.4	193.5	154	145
17	3	HDPE	10	2.3	191.5	89	78
18	3	HDPE	30	6.5	193.5	125	110
19	3	HDPE	50	9.2	198.5	139	123
20	3	HDPE	70	12.5	200.6	153	136
21	3	HDPE	90	15.5	200.6	164	146
22	3	HDPE	110	18.2	200.1	172	155
23	3	HDPE	130	22.6	200.1	183	169
24	3	HDPE	150	27.6	198.5	195	177
25	3	PP-R	10	2.4	209.7	13	10
26	3	PP-R	30	7.0	213.3	26	18
27	3	PP-R	50	10.9	213.6	32	26
28	3	PP-R	70	14.6	211.1	38	35
29	3	PP-R	90	17.7	209.6	42	40
30	3	PP-R	110	22.4	209.2	48	45
31	3	PP-R	130	25.3	208.8	51	47
32	3	PP-R	150	28.6	207.9	54	49
33	4	PP-R	10	2.2	207.7	20	22
34	4	PP-R	30	5.6	210.0	46	47
35	4	PP-R	50	9.6	211.2	58	58
36	4	PP-R	70	13.2	210.0	70	70
37	4	PP-R	90	16.0	212.1	79	78
38	4	PP-R	110	19.9	215.2	87	84
39	4	PP-R	130	21.7	218.6	97	93
40	4	PP-R	150	25.5	220.8	102	98

**Table A2:** Nomenclature.

*a*	width of transition	*n_p_*	power law index
*a_s_*	amplitude of sine	*N*	screw speed
*a_t_*	temperature-shift factor	*N_z_*	number of downchannel elements
***D***	rate of deformation tensor	*p*	pressure
*D_b_*	barrel diameter	***p***	pressure vector
*f_p_*	correction factor	*t*	flight width
*h*	channel height	*T*	temperature
*h_v_*	channel height in the wave valley	*T*_0_	reference temperature
*h_p_*	channel height in the wave peak	***v***	velocity vector
*i*	index of network elements	*w*	channel width
*k*	linearized die conductance	*z*	downchannel coordinate
***k***	conductance matrix	*α*	temperature coefficient
*K*	consistency	*δ*	flight clearance
*K’*	die conductance in nonlinear die equation	$\dot{\gamma }$	shear rate
*L*	axial length	*η*	viscosity
***L***	velocity gradient tensor	*η_c_*	viscosity (Carreau–Yasuda model)
*m*	flow exponent	*η_p_*	viscosity (power law model)
*ṁ*	mass flow rate	*η_0_*	zero-shear viscosity
***ṁ***	mass flow vector	*η_∞_*	infinite-shear viscosity
*ṁ_d_*	theoretical drag flow	*λ*	characteristic relaxation time
***ṁ_d_***	drag flow vector	*ρ_m_*	melt density
*ṁ_p_*	pressure flow	***τ***	stress tensor
*n*	number of waves	*Φ*	fluidity
*n_c_*	power law index (Carreau–Yasuda model)		
